# Formation of Adaptive Trophic Niches of Euryphagous Fish Species in Response to Off-Seasonal Water Level Regulation in Hongze Lake

**DOI:** 10.3390/ani15010059

**Published:** 2024-12-30

**Authors:** Si Luo, Zexin Wang, Shengyu Zhang, Huan Mu, Yubin Jiao, Xiao Qu, Qishuo Wang, Ruiqi Yang, Yanxia Zuo, Shiyu Jin

**Affiliations:** 1 School of Life Science and Food Engineering, Huaiyin Institute of Technology, Huai’an 223003, China; luosi@hyit.edu.cn (S.L.); 18303615694@163.com (Z.W.); jiaoyubinmail@163.com (Y.J.); 13258004185@163.com (R.Y.); 2 Hongze Lake Fisheries Management Committee Office, Huai’an 223003, China; hzhzsy@126.com (S.Z.); hawmu110@126.com (H.M.); 3 State Key Laboratory of Freshwater Ecology and Biotechnology, Institute of Hydrobiology, Chinese Academy of Sciences, Wuhan 430072, China; quxiao@hainanu.edu.cn (X.Q.); wangqishuo@ihb.ac.cn (Q.W.); yxzuo@ihb.ac.cn (Y.Z.); 4 School of Marine Biology and Fisheries, Hainan University, Haikou 570228, China

**Keywords:** SEAc, niche overlap, water level fluctuations, species coexistence, stable isotope analysis

## Abstract

Off-seasonal water level regulations disrupt the biological traits and phenological rhythms of native fish species, yet their impacts on interspecific trophic interactions remain understudied. This study employed stable isotope analysis to assess the trophic dynamics of three fish species (*Parabramis pekinensis*, *Carassius auratus*, and *Toxabramis swinhonis*) across different water periods in Hongze Lake. The findings revealed that all three species occupied similar mid-level trophic positions, with no significant difference among water periods (*p* > 0.05). During high-water periods, *P. pekinensis* and *T. swinhonis* exploited broader niches, while *C. auratus* relied on a narrower diet. In contrast, during low-water periods, *C. auratus* expanded its niche, while *P. pekinensis* and *T. swinhonis* reduced their isotopic niche widths. Niche overlap analysis showed minimal trophic overlap among the three species during high-water periods, with increased overlap during low-water periods, except for the highest overlap between *C. auratus* and *T. swinhonis* during mid-water periods. This variation in niche overlap aligns with shifts in dietary reliance, as POM was the predominant dietary component for all three species, but its contribution varied significantly across different water periods. These findings indicated that adaptive trophic niche facilitated the coexistence of these fish species, while off-seasonal water level regulation may intensify interspecific competition. These insights are essential for refining water management policies and developing sustainable fishery management strategies of Hongze Lake and other water-level-regulated systems.

## Simple Summary

Hongze Lake, a key storage lake in China’s South-to-North Water Diversion project, experiences off-seasonal water level regulation that disrupts native fish and others. This study explored the trophic dynamics of three fish species—*Parabramis pekinensis*, *Carassius auratus*, and *Toxabramis swinhonis*—using stable isotope analysis during high-, mid-, and low-water periods. Results showed that all three fish species generally occupied similar mid-level trophic positions across different water periods. The SEAc values of *P. pekinensis* and *T. swinhonis* were highest during high-water periods and lowest during low-water periods, whereas *C. auratus* exhibited the opposite trend. These findings suggest that *P. pekinensis* and *T. swinhonis* function as optimal foragers, while *C. auratus* adopts a generalist strategy, consistent with classical competition theory. Niche overlap analysis demonstrated that dynamic resource partitioning during high-water periods and resource sharing during low-water periods promote the coexistence of these species. This coexistence strategy is further influenced by shifts in dietary composition, as although POM was the main dietary component, its contribution decreased over time as SOM and macrophytes became increasingly significant, highlighting the adaptability of these species to fluctuating environments. This study emphasizes the role of water level fluctuations in shaping interspecific interactions, providing insights into coexistence mechanisms of euryphagous fish species and ecological dynamics in Hongze Lake.

## 1. Introduction

Large-scale inter-basin water diversion projects have been implemented globally to address water scarcity issues, yet they often induce substantial hydrological changes in lakes along their routes, resulting in water level fluctuations that diverge sharply from natural patterns [1,2]. A prevalent method of water level management in these systems is off-seasonal regulation, wherein water levels are lowered during the wet season to facilitate flood control and raised during the dry season to support various demands, including water supply, irrigation, and navigation. This management strategy causes water level fluctuations that are misaligned with local precipitation patterns and the natural flow-regime timing of lakes. These fluctuations, now the dominant force shaping the structure and function of lake ecosystems along the diversion routes, effectively enhance flood control and secure water supply, irrigation, and navigation during dry seasons [2,3]. However, they also disrupt the biological traits, and phenological rhythms of local fish species, potentially leading to profound impacts on aquatic ecosystems [4,5,6].

Feeding is a fundamental biological function in fish, playing a vital role in material cycling and energy flow within aquatic ecosystems. Food resources are a primary driver of ecological segregation among fish species, facilitating the efficient resource exploitation and promoting species coexistence [7]. Water level fluctuations can impact these dynamics significantly [8]. During low-water periods, the reduction in lake area and volume, coupled with decreased connectivity to adjacent water bodies, confines fish to smaller habitats, intensifying abiotic stress and competition [9]. Even slight decrease in water levels in lakes can significantly diminish available feeding habitats [10], further heightening competition [11]. Additionally, water level fluctuation can alter food resources availability, indirectly influencing fish feeding strategies [12,13]. For instance, Hansen et al. [6] found that prolonged declines in water levels reduced food diversity by up to 46%, forcing fish to shift their diets toward alternative food resources. Conversely, high-water periods connect diverse hydrological environments, expand foraging areas, and are expected to mitigate trophic interactions due to the influx of allochthonous food resources and increased habitat diversification [14].

In general, fish have evolved to adapt to natural water level changes and achieve sympatric species coexistence through trophic, spatial, or temporal segregation, particularly under resource-limited conditions [15,16]. However, the extent to which trophic niches expand or contract in response to water level fluctuation cannot be universally generalized. According to Optimal Foraging Theory, species tend to become more specialized when preferred resources are abundant and broaden their diets when food is scarce [17,18]. Such strategies have been observed in *Coreius heterodon* (Bleeker, 1864) and *Rhinogobio ventralis* (Sauvage and Dabry de Thiersant, 1874) in the upper Yangtze River [19], *Carassius auratus* (Linnaeus, 1758) in Poyang Lake [20], and frugivorous fishes in the Amazon basin [8] during high- and low-water periods. In contrast, Competition Theory and Niche Partitioning [8,21,22] suggest that species may broaden their diets, leading to increased niche overlap when resources are abundant, while narrow their dietary breadth to reduce interspecific competition during the periods of resource scarcity. Such contrasting feeding strategies have been observed in *Prochilodus nigricans* (Spix and Agassiz, 1829), *Cichla pleiozona* (Kullander and Ferreira, 2006) and *Serrasalmus rhombeus* (Linnaeus, 1766) in an Amazonian floodplain lake [23], *Moenkhausia forestii* (Benine, Mariguela and Oliveira, 2009) in the Upper Paraná River floodplain [24], and *Anabas testudineus* (Bloch, 1792), *Boesemania microplepis* (Bleeker, 1858) and *Notopterus notopterus* (Pallas, 1769) in the Tonle Sap Lake [25]. While fish have developed diverse feeding strategies to adapt to natural water level fluctuations, the ecological consequences of unnatural water level fluctuations causing by water diversion projects remain poorly understood.

China’s South-to-North Water Diversion Project, one of the largest water diversion projects in the world, plays a critical role in relieving water shortages and supporting economic and social development in northern China [5]. The project comprises eastern, central, and western routes, with Hongze Lake, China’s fourth-largest freshwater lake, serving as the largest regulatory along the eastern route [3]. The health of its ecosystem is vital not only to the economic sustainability of the northern Jiangsu region but also to the overall success of the diversion project. The intensive water level regulation associated with this project has caused significant changes in the hydrological regime of Hongze Lake, leading to pronounced off-seasonal water level fluctuation [26]. Typically, the lake experiences relatively high-water levels from January to April, peaking in March (high-water periods) [26]. The water levels then decrease rapidly, remaining low until August (low-water periods), rising again in September and maintaining elevated levels thereafter (high-water period) [26]. Recent surveys have documented notable shifts in the fish community structure of Hongze Lake, revealing a decline in biodiversity and a growing dominance of small omnivorous fish species [27,28,29]. Among these, three omnivorous fish species, *Parabramis pekinensis* (Basilewsky, 1855), *C. auratus* and *Toxabramis swinhonis* (Günther, 1873), are particularly dominant [28], sharing significant dietary overlap [30,31,32]. Among them, *T. swinhonis* is a small fish of low economic value, whereas *C. auratus* and *P. pekinensis* are economically significant species in Hongze Lake. Interestingly, the density and biomass of their prey such as zooplankton, phytoplankton, and macrophytes exhibited contrasting trends during high- and low-water periods in Hongze Lake, being higher during low-water periods [33,34,35]. This scenario provides a valuable opportunity to assess the impacts of off-seasonal water level regulation on interspecific trophic dynamics and the associated ecological adaptation mechanisms of these species in Hongze Lake.

In this study, we hypothesize that the isotopic niche width and interspecific niche overlap of the three fish species in Hongze Lake respond dynamically to habitat and food resources changes driven by off-seasonal water level regulation. Specifically, we test the following hypotheses: (1) niche width and overlap decrease during low-water periods and increase during high-water periods, as predicted by Optimal Foraging Theory; (2) niche width and overlap increase during low-water periods and decrease during high-water periods, as suggested by Competition Theory and Niche Partitioning. To test these hypotheses, we employ stable isotope analysis to quantify the trophic positions of each species, compare their isotopic niche width and temporal niche dynamics, and assess resource partitioning and utilization through interspecific isotopic niche overlap and the relative contributions of primary carbon sources. The objectives of the present study are to: (1) reveal the ecological mechanisms underpinning resource partitioning and coexistence among the omnivorous fish species in response to fluctuating environmental conditions; (2) provide novel insights into the ecological impacts of off-seasonal water level regulation on storage lake ecosystems. The findings could provide scientific basis for water level regulation and fishery management of Hongze Lake and other storage lakes.

## 2. Materials and Methods

### 2.1. Study Area and Sampling Procedure

Hongze Lake (33°06′–33°40′ N, 118°10′–118°52′ E), located in the middle and lower reaches of the Huai River, spans the Huai’an and Suqian regions of Jiangsu Province and covers a catchment area of 158,000 km^2^. The lake serves as a critical water storage reservoir and a major conduit for water transfer, playing a vital role in regional flood management, water supply, irrigation, transportation, and biodiversity conservation. The lake’s flood control level is maintained at 12.5 m during the flood season, with a normal water level of 13.5 m during the non-flood season, and a design flood level (maximum water level during the design flood) of 16.0 m [26]. The effective reservoir capacity is 2.4 billion m^3^, while the flood regulation capacity is 9.8 billion m^3^. The maximum and average water depths are 4.5 m and 1.9 m, respectively [36]. The primary outlets include the Inflow Channel to the Yangtze River, which directs water from Hongze Lake into the Yangtze River, the North Jiangsu Irrigation Canal, Huaishuxinhe River, and the channel leading to the Yellow Sea. Situated in a warm temperate zone, Hongze Lake experiences a semi-humid monsoon climate [36]. The average annual air temperature in the Hongze area was 15.1 °C, with an average annual precipitation of approximately 1027 mm. Seasonal variations include an average winter temperature of 2.93 °C and a summer temperature of 26.27 °C, with 57.08% of the annual precipitation occurring in summer and only 7.01% in winter.

**Figure 1:**
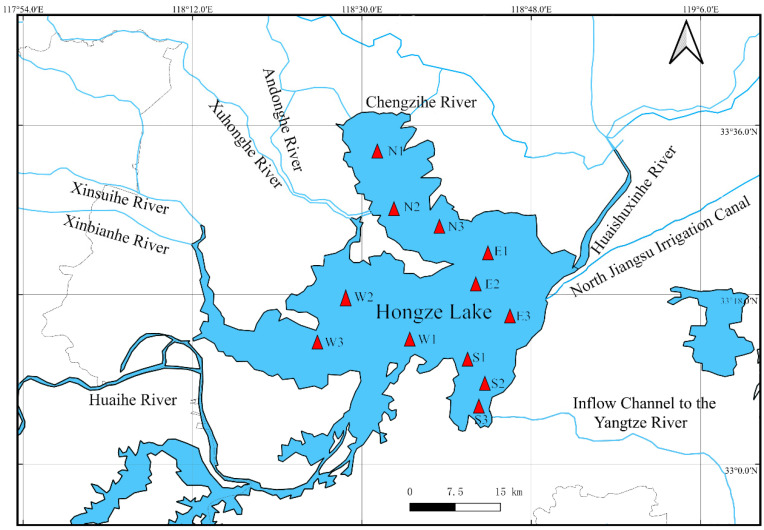
Sketch map of Hongze Lake and the distribution of sampling sites. N1–N3: Sampling sites in the northern lake region; E1–E3: Sampling sites in the eastern lake region; S1–S3: Sampling sites in the southern lake region; W1–W3: Sampling sites in the western lake region.

**Figure 2:**
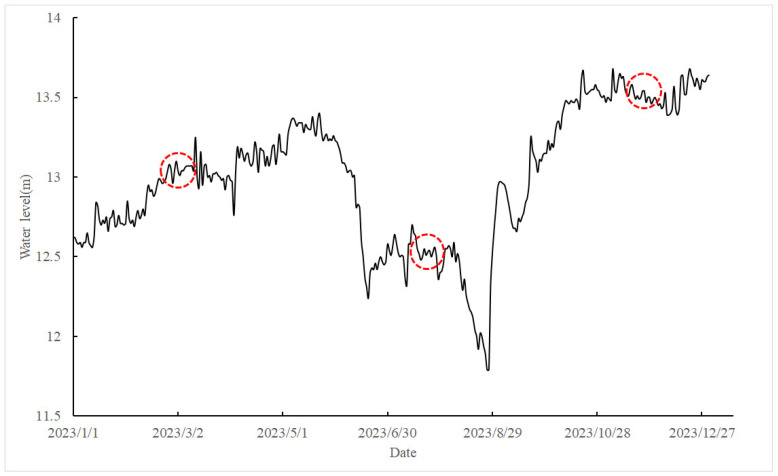
Water level fluctuation of Hongze Lake in 2023 (red dashed circle: sampling time).

In this study, the lake was divided into four distinct regions—eastern, western, southern and northern—and 12 sampling stations were selected based on the lake’s morphological features (Figure 1). Field sampling was conducted in Hongze Lake during March 2023 (mid-water periods), July 2023 (low-water periods), and November 2023 (high-water periods) (Figure 2). Primary carbon sources, including sedimentary organic matter (SOM), particulate organic matter (POM), macrophyte, along with three fish species (*P. pekinensis*, *C. auratus* and *T. swinhonis*) were chosen for stable isotope analysis. Sediment samples was collected using a grab sediment sampler, with SOM samples extracted from the top 1 cm of the sediment surface [37,38]. POM was obtained by pre-filtering 10 L of surface water through a 64 µm plankton net, followed by filtration through 200 µm mesh sieves to remove large inorganic particles and zooplankton [37,38]. The pre-filtered water was then passed through pre-combusted Whatman GF/F glass fiber filters (heated at 450 °C for 4 h to remove organic matter) using a pump system [37,38]. The filters were wrapped in tin foil and stored at −20 °C for subsequent stable isotope analysis. Given that the target fishes predominantly fed on submerged plants, *Potamogeton crispus* (Linnaeus, 1753), *Elodea nuttallii* ((Planch.) H.St.John, 1920), *Myriophyllum spicatum* (Linnaeus, 1753), and *Ceratophyllum demersum* (Linnaeus, 1753) were selected for isotopic analysis. Macrophyte were rinsed with distilled water to remove any attached organisms, sediments and other debris, and then wrapped in tin foil for further analysis. Shellfish species, including *Corbicula fluminea* (Müller, 1774), *Cristaria plicata* (Leach, 1815), *Arconaia lanceolate* (Lea, 1856), and *Hyriopsis cumingii* (Lea, 1852) were also collected. The shells and stomachs were removed, and the internal tissues were isolated for analysis. Fish were captured using gillnets, trawl nets, and cylindrical traps, and white muscle tissue near the first dorsal fin was extracted, rinsed with distilled water to remove exogenous materials such as scales or bones.

Water samples were collected simultaneously with fish sampling at the same sites, and a set of 9 physico-chemical water quality variables related to water level fluctuation were monitored and analyzed (Table 1). Water temperature, conductivity, dissolved oxygen, and pH were monitored using a portable multi-parameter water quality meter (YSI Professional Plus). Water depth and transparency were assessed by a Secchi disk. At each site, 1 L water sample was collected and immediately transported in a cooler with ice to laboratory for analysis. The total phosphorus, total nitrogen, and chlorophyll a were analyzed according to APHA [39].

### 2.2. Stable Isotope Analysis

All samples were dried at 60 °C for 48 h until a constant weight was reached. The dried samples were then finely ground using a high-throughput tissue grinder (Bionoon-48F, Bionoon, Shanghai, China). Subsequently, processed samples weighing between 300 and 500 µg were placed into tin capsules for analysis. The analysis using an EA-HT Elemental Analyzer (Thermo Fisher Scientific, Inc., Bremen, Germany) coupled with an Isotope Ratio Mass Spectrometer (Delta V Advantage, Thermofisher, Rheinfelden, Germany) at the Institute of Hydrobiology, Chinese Academy of Sciences, Wuhan. For glass fiber filter membrane samples, the material was meticulously scraped off the filters and loaded into tin capsules for stable isotope analysis. The samples were combusted in the EA-HT Elemental Analyzer to produce CO_2_ and N_2_ gases, which were then analyzed by the Isotope Ratio Mass Spectrometer. The spectrometer measured the ratio of ^13^C to ^12^C in the CO_2_, comparing it to an international standard (Pee Dee Belemnite or PDB) to caculate the δ^13^C value for each sample. Similarly, the ratio of ^15^N to ^14^N was compared to the international standard (pure N_2_ in air) to determine the δ^15^N value. The international standard materials used were IAEA-USGS40 and IAEA-USGS41. The isotope values for δ^15^N (‰) and δ^13^C (‰) were according to the following equation:

δ^13^C (‰) = [(*R_sample_*/*R_standard_*) − 1] × 1000

δ^15^N (‰) = [(*R_sample_*/*R_standard_*) − 1] × 1000.

### 2.3. Trophic Position Calculation

The R package tRophicPosition was applied to calculate the TP of three fish species during different water periods. This package implements three Bayesian models based on the number of baselines, and incorporating individual variability while also accounting for sampling error in trophic discrimination factors (TDF), baselines and higher consumers, yielding posterior estimates of parameters [40]. The δ^15^N and δ^13^C values of consumers, baselines and TDF are modeled as random variables, each with a prior normal distribution on their means and a uniform prior distribution on their standard deviations. Finally, the TPs for each fish species are modeled using the following two equation:

δ^15^N_c_ = ΔN(*TP* + ƛ) + α(δ^15^N_b1_ + δ^15^N_b2_) − δ^15^N_b2_

δ^13^C_c_ = δ^13^C_b1_α + δ^13^C_b2_(1 − α)

α = ((δ^13^C_b2_ − (δ^13^C_c_ + ΔC))/(*TP* − ƛ))/(δ^13^C_b2_ + δ^13^C_b1_)

where δ^15^N_c_, δ^13^C_c_, δ^15^N_b1_, δ^15^N_b2_, δ^13^C_b1_, and δ^13^C_b2_ refer to the δ^15^N and δ^13^C values of consumers, baselines 1 and 2, respectively, α is the proportion of N derived from baseline 1, and λ is the trophic position of the baseline [41].

### 2.4. Isotopic Niche Width and Elllipses Overlap

Traditionally, six niche/community metrics have been used to describe ecological niches, ranging from individual organisms to entire communities [42]. These metrics include: δ^15^N range (NR), which provides information on the trophic length of the community; δ^13^C range (CR), which estimates the diversity of primary carbon sources; total area of the convex hull encompassing the data points (TA), indicating niche width; mean distance to centroid (CD), offering additional insights into niche width as well as species spacing; mean nearest neighbor distance (MNND), measuring species density and clustering within the community; and standard deviation of the nearest neighbor distance (SDNND), assessing the evenness of spatial density and packing. Among these, TA has been the most widely used metric to describe the niche width of species or communities. However, a significant limitation of these metrics is their sensitive to sample size, particularly when sample sizes are small (*n* < 50), a common occurrence in ecological studies. This sensitivity poses challenges when sample sizes vary within a study or when comparisons are made across multiple studies. Additionally, when applied to an entire community, these metrics fail to incorporate the natural variability within the system, resulting in summary statistics that provide only a point estimate of each metric.

In this study, the isotopic niche width of the three fish species was initially estimated using the standard ellipse area (SEA), calculated with the SIBER package in R [42]. The SEA is analogous to the standard deviation for bivariate data, with the shape and size of the ellipses defined by the covariance matrix of δ^13^C and δ^15^N, and their position determined by the means of these variables. To mitigate potential bias due to small sample size, the SEA was corrected for sample size (SEAc), making it insensitive to such bias [42]. The SEAc was fitted to bivariate data using maximum likelihood estimators, with the isotopic niche width expressed as the SEAc in‰^2^. The SEAc encompassed approximately 40% of the data, providing robust estimates of isotopic niche width for direct comparison across different sample sizes, while excluding extreme values that could distort the total niche area [43].

When performing the analysis in R, the first step involved fitting Bayesian multivariate normal distributions to each group within the dataset. Once they were fitted to each group, the SEA was calculated from the posterior distribution of the covariance matrix for each group, resulting in the SEA and SEAc. In this study, two sets of standard ellipses were generated using the SIBER package. The first set depicted the isotopic niche of three fish species in Hongze Lake, with δ^13^C and δ^15^N values from all sampling locations and all water periods (mid-water period, low-water period and high-water period) inputted to generate ellipses for each species. The second set showed the isotopic niche of three fish species during each water period, with δ^13^C and δ^15^N values from all sampling locations during each water period combined to generate nine ellipses for each species.

To compare the isotopic niche sizes of the three fish species, Bayesian SEA (SEAB) was estimated using Markov chain Monte Carlo (MCMC) simulations to construct the posterior estimates. Pairwise comparisons of niche width between fish species were conducted to determine the probability of differences between niche areas using Bayesian inference. To test whether one group’s ellipse was smaller or larger than another, the probability that its posterior distribution was smaller or larger was calculate by comparing each pair of posterior draws for both groups and determining which was smaller in magnitude.

Additionally, overlap between two or more groups was estimated as an indicator of similarity and differences in isotopic niche space utilization. The overlap, ranging from 0% to 100%, represents the fraction of overlap between the SEAc of two groups.

### 2.5. Statistical Analysis

Kruskal–Wallis tests were conducted to assess differences in isotopic signatures among fish species and primary carbon sources, with pairwise Wilcoxon post hoc tests applied for further comparisons. To evaluate whether isotopic signatures varied across water periods and species (including fish and primary carbon sources), a permutation multivariate analysis of variance (PERMANOVA) based on Euclidean distance was employed. To estimate the contribution of primary carbon sources to the diet of each fish species, Bayesian stable isotope mixing models from the ‘SIAR’ package in R were employed [44]. This model approach offers robust statistical power by incorporating uncertainties in the sources, the consumers’ isotopic signatures and the fractionation values. Fraction factor of 3.4‰ for δ^15^N and 0.8‰ for δ^13^C were applied, following the recommendations from a previous study [45]. All analyses were performed by R version 3.3.2 [46], and the significance level was set to 0.05.

## 3. Results

### 3.1. Carbon and Nitrogen Isotopes of Primary Carbon Sources and Three Fish Species

A total of 17 POM, 17 SOM, 9 macrophyte and 180 fish samples—with 60 samples each from *P. pekinensis*, *C. auratus* and *T. swinhonis*—were collected in Hongze Lake during three different water periods and analyzed for carbon and nitrogen isotopic ratios. The body length and body weight of *P. pekinensis* during the mid-, low-, and high-water periods were 23.04 ± 2.98 cm and 229.47 ± 89.22 g, 21.69 ± 2.65 cm and 159.64 ± 90.82 g, 21.98 ± 4.11 cm and 190.43 ± 128.02 g, respectively. For *C. auratus*, the body length and body weight during these periods were 17.46 ± 2.27 cm and 201.46 ± 8.44 g, 15.33 ± 1.48 cm and 116.74 ± 27.44 g, 18.96 ± 3.01 cm and 229.30 ± 100.77 g, respectively. The body length and body weight of *T. swinhonis* sampled during the three water periods were 10.65 ± 0.95 cm and 13.91 ± 4.28 g, 11.69 ± 0.89 cm and 15.74 ± 3.27 g, 11.15 ± 1.88 cm and 15.37 ± 6.49 g, respectively.

**Figure 3:**
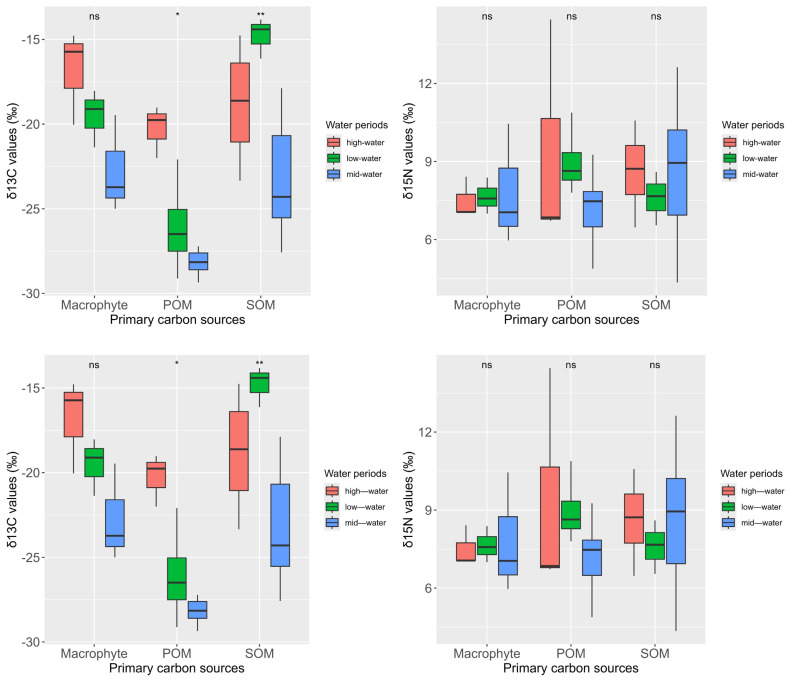
Boxplot of δ^13^C and δ^15^N values for primary carbon sources (macrophyte, POM: particulate organic matter, and SOM: sedimentary organic matter) in Hongze lake. Box-plot representation: the horizontal line inside the box represents the median, and the lower and upper borders of the box represent the 25th and 75th percentiles, respectively. The upper and lower whiskers indicate the maximum and minimum range of the data excluding outliers. ns: no significant differences in the δ^13^C or δ^15^N values among water periods; *: *p* < 0.05; **: *p* < 0.01.

The isotopic signatures of the primary carbon sources (SOM, POM, and macrophyte) displayed significant variability (Figure 3). δ^13^C values ranged from −29.35‰ to −13.82‰, while δ^15^N spanned from 4.34‰ to 14.46‰. Specifically, the δ^13^C value of POM (−26.29 ± 3.37‰) was significantly lower than those of SOM (−20.82 ± 4.59‰) and macrophyte (−19.70 ± 3.36‰) (Kruskal-wallis test and pairwise Wilcoxon test, χ^2^ = 17.263, *df* = 2, POM vs. macrophyte: *p* = 0.0003, POM vs. SOM: *p* = 0.0002; macrophyte vs. SOM: *p* = 0.5967). In contrast, δ^15^N values did not exhibit significant differences among the carbon sources (Kruskal-wallis test, χ^2^ = 1.416, *df* = 2, *p* = 0.4927). Seasonal patterns in δ^13^C values revealed an enrichment trend from mid-water periods to high-water periods, with significant differences between mid-water periods and both low-water periods and high-water periods (pairwise Wilcoxon test, mid-water periods vs. low-water periods: *p* = 0.0160, mid-water period vs. high-water periods: *p* < 0.0001; high-water periods vs. low-water periods: *p* = 0.5290). Although δ^15^N values varied seasonally (mid-water period: 4.35‰ to 12.63‰, low-water periods: 6.55‰ to 10.88‰, high-water periods: 6.47‰ to 14.46‰), these differences were not statistically significant.

**Figure 4:**
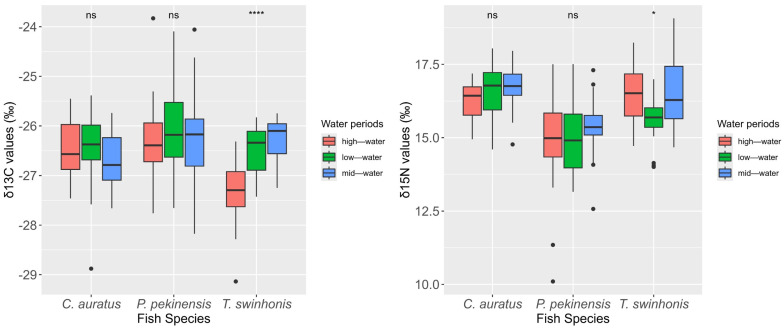
Boxplot of δ^13^C and δ^15^N values for *Carassius auratus*, *Parabramis pekinensis*, and *Toxabramis swinhonis* in Hongze lake. Box-plot representation: the horizontal line inside the box represents the median, and the lower and upper borders of the box represent the 25th and 75th percentiles, respectively. The upper and lower whiskers indicate the maximum and minimum range of the data excluding outliers (black dots). ns: no significant differences in the δ^13^C or δ^15^N values among water periods; *: *p* < 0.05; ****: *p* < 0.001.

The three fish species in Hongze Lake displayed significant variations in both δ^13^C and δ^15^N values (Figure 4, Kruskal–Wallis test, δ^13^C: χ^2^ = 8.9491, *df* = 2, *p* < 0.001; δ^15^N: χ^2^ = 13.083, *df* = 2, *p* < 0.001). The δ^13^C values ranged from −29.14‰ to −23.83‰, while the δ^15^N values spanned from 10.10‰ to 19.07‰. Specifically, the δ^13^C values of *P. pekinensis* (mean ± SE, −26.22 ± 0.12‰) were significantly higher than those of *C. auratus* (−26.54 ± 0.08‰), and *T. swinhonis* (−26.72 ± 0.09‰) (pairwise Wilcoxon test, *P. pekinensis* vs. *C. auratus*: *p* = 0.0447, *P. pekinensis* vs. *T. swinhonis*: *p* = 0.0048; *C. auratus* vs. *T. swinhonis*: *p* = 0.2345). In contrast, the δ^15^N values of *P. pekinensis* (mean ± SE, 15.06 ± 0.17‰) were significantly lower than those of *C. auratus* (16.49 ± 0.10‰) and *T. swinhonis* (16.23 ± 0.14‰) (Kruskal-wallis test and pairwise Wilcoxon test, χ^2^ = 44.403, *df* = 2, *P. pekinensis* vs. *C. auratus*: *p* < 0.001, *P. pekinensis* vs. *T. swinhonis*: *p* < 0.001; *C. auratus* vs. *T. swinhonis*: *p* = 0.063).

The PERMANOVA test, based on Euclidean distance, identified significant differences in isotopic signatures across different water periods and among fish species (water periods: *F* = 3.7622, *df* = 2, *p* = 0.008; Species: *F* = 22.3830, *df* = 2, *p* = 0.001), with notable interactions between water periods and species (*F* = 2.8193, *df* = 4, *p* = 0.018). Although δ^15^N values were higher in mid-water periods for all species compared to low-water periods and high-water periods, the differences between water periods were not significant (mid-water periods vs. low-water periods: *p* = 0.069; high-water periods vs. low-water periods: *p* = 0.330; high-water periods vs. mid-water periods: *p* = 0.252). Significant differences in isotopic signatures were observed between *P. pekinensis* and *C. auratus* (*p* = 0.003), *P. pekinensis* and *T. swinhonis* (*p* = 0.003), while no significant differences were found between *C. auratus* and *T. swinhonis* (*p* = 0.396).

### 3.2. Trophic Position of the Three Fish Species

**Figure 5:**
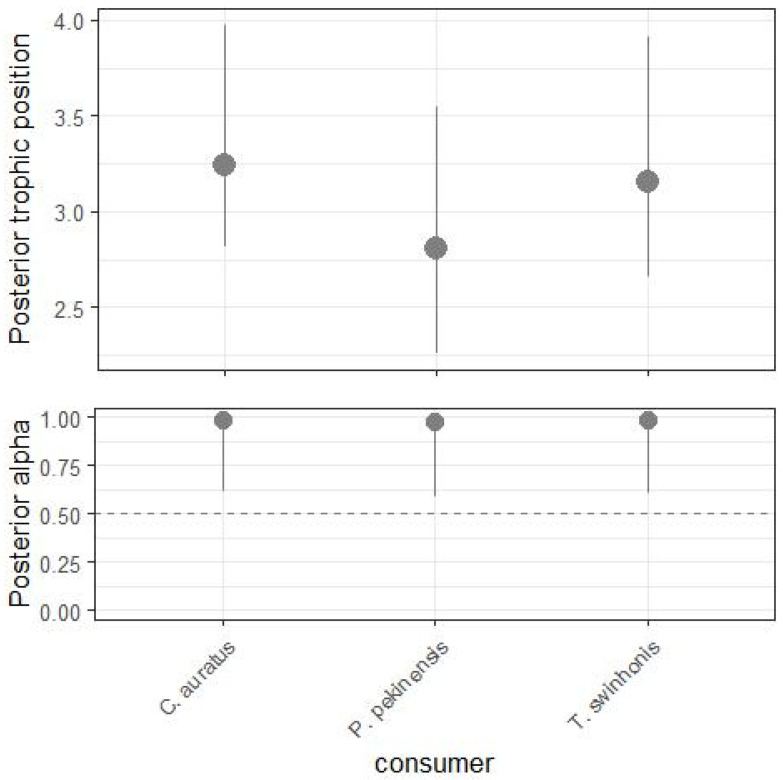
Bayesian posterior trophic positions for *Carassius auratus*, *Parabramis pekinensis*, *Toxabramis swinhonis* and posterior α indicating the relative contribution of each baseline to the energy inputs of each species in Hongze lake. Overlap of the 95% credible intervals indicates similarity between groups.

*P. pekinensis*, *C. auratus*, and *T. swinhonis* generally occupied similar mid-level TPs. The mode of the Bayesian estimated TPs (with 95% credibility intervals) were 2.795 (2.356–3.516) for *P. pekinensis*, 3.251 (2.788–4.683) for *C. auratus*, and 3.181 (2.830–4.650) for *T. swinhonis* (Figure 5). According to the Bayesian posterior distributions, there were no significant differences in TPs among the three species (*p* > 0.05). Although *P. pekinensis* exhibited a slightly lower TP compared to *C. auratus* and *T. swinhonis*, these differences were not statistically significant, indicating that these species likely fulfill similar functional roles within Hongze Lake.

**Figure 6:**
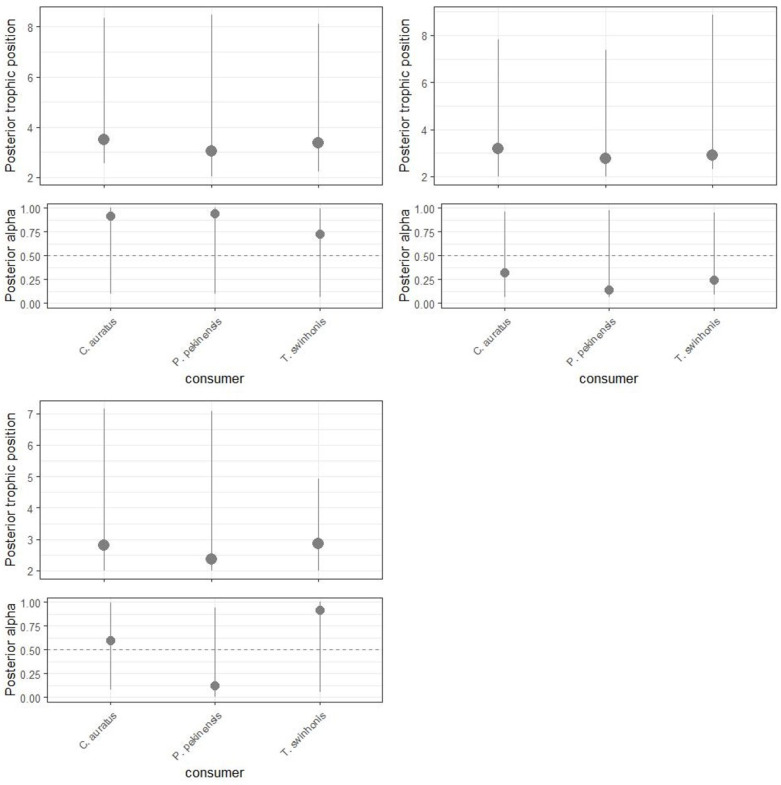
Bayesian posterior trophic positions and posterior α values (relative contribution of each baseline to the energy inputs of each species) for *Carassius auratus*, *Parabramis pekinensis*, *Toxabramis swinhonis* during high-, low-, and mid-water periods of Hongze lake. Overlap of the 95% credible intervals indicates similarity between groups.

The TPs of all three fish species varied across different water periods, with a general decline observed from mid-water periods to high-water periods. For *P. pekinensis*, the modes of the Bayesian estimated TPs (95% credibility intervals) were 3.019 (2.266–9.120) during mid-water periods, 2.735 (2.184–7.708) during low-water periods, and 2.39 (2.059–7.583) during high-water periods. For *C. auratus*, the TPs were 3.508 (2.466–7.781) during mid-water periods, 3.16 (2.315–8.562) during low-water periods, and 2.812 (2.184–8.107) during high-water periods. For *T. swinhonis*, the values were 3.383 (2.535–9.441) during mid-water periods, 2.908 (2.282–8.375) during low-water periods, and 2.877 (2.187–6.369) during high-water periods (Figure 6). However, there were no significant differences in TPs among the three fish species across the different water periods (*p* > 0.05).

### 3.3. Isotopic Niche Width, Ellipses Overlap and Contribution of Primary Carbon Sources to the Diets of Three Fish Species

**Figure 7:**
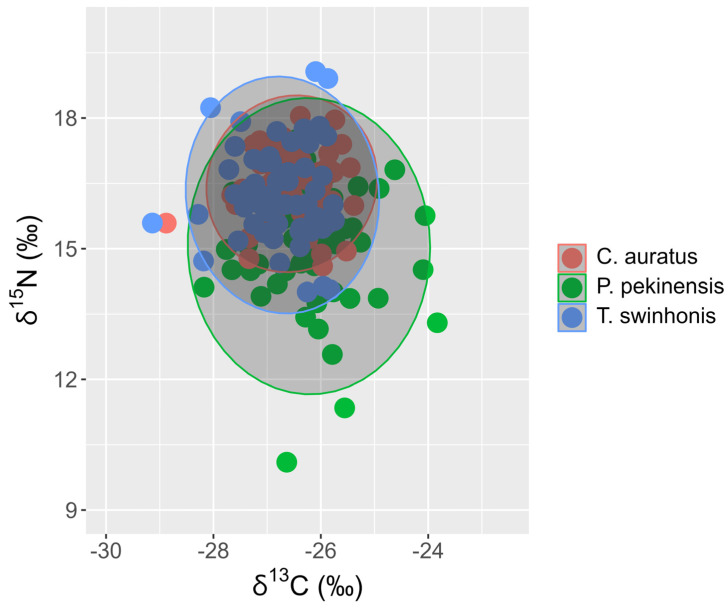
Individual δ^13^C and δ^15^N values with the corresponding standard ellipses area (SEAc, ‰^2^) for the three fishes (*Carassius auratus*, *Parabramis pekinensis*, and *Toxabramis swinhonis*) in Hongze Lake.

**Figure 8:**
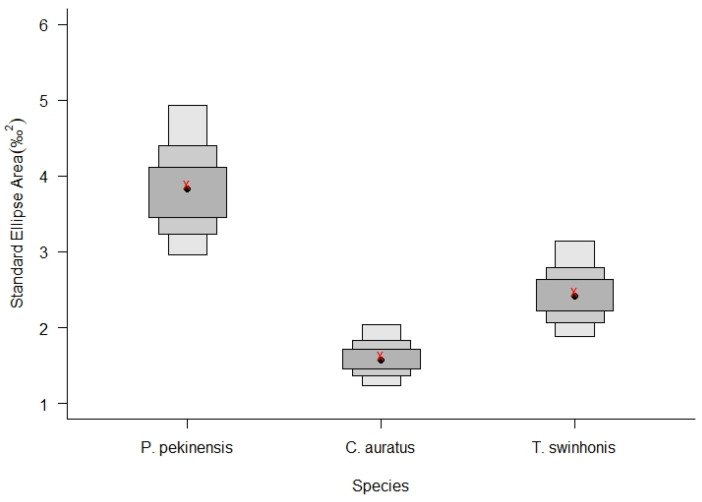
The posterior distribution of Bayesian-based Standard Ellipse Areas (SEAB) with 50%, 75%, and 95% credibility intervals for the three fishes (*Carassius auratus*, *Parabramis pekinensis*, and *Toxabramis swinhonis*) in Hongze Lake. Crosses in red represent the area of the standard ellipse (SEAc). Red x: the estimated SEAc, black dots: SEAB mode.

The analysis of isotopic niche widths (SEAc) and overlap (95% Bayesian ellipses) among trophic groups demonstrated variability across the species in Hongze Lake. *P. pekinensis* exhibited the broadest isotopic niche (mean SEAc and 95% credibility interval: 3.8924‰^2^, 2.9336–4.8709‰^2^), followed by *T. swinhonis* (2.4857‰^2^, 1.8666–3.1352‰^2^), and *C. auratus* (1.6338‰^2^, 1.2228–2.0417‰^2^) (Figure 7 and Figure 8). SIBER estimations from the maximum likelihood estimated ellipses using Bayesian models indicated strong competitive interactions for resources among the three fish species in Hongze Lake. The highest niche overlap occurred between *C. auratus* and *T. swinhonis* (39.52%, Table 2), with a lower overlap observed between *P. pekinensis* and *C. auratus* (28.79%, Table 2).

**Figure 9:**
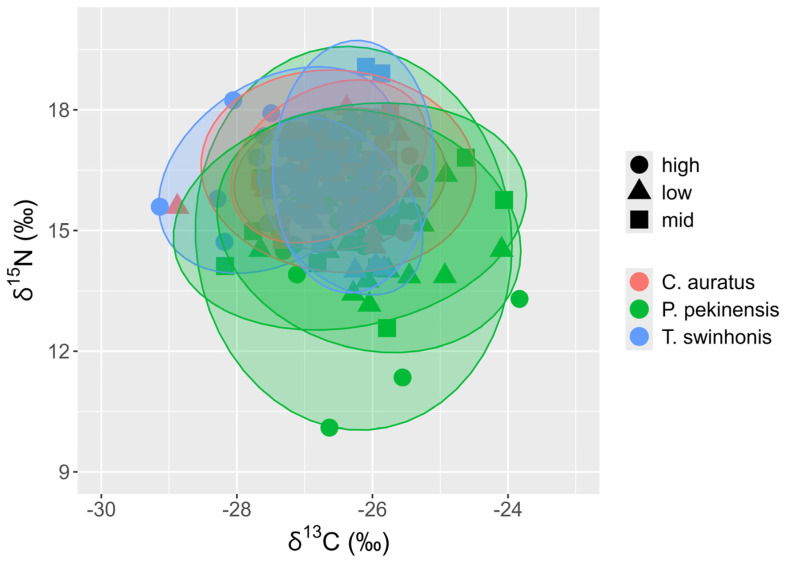
Individual δ^13^C and δ^15^N values with the corresponding standard ellipses area (SEAc, ‰^2^) for each fish (*Carassius auratus*, *Parabramis pekinensis*, and *Toxabramis swinhonis*) across different water periods.

**Figure 10:**
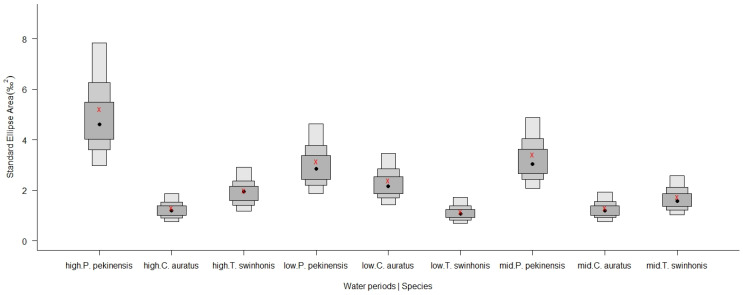
The posterior distribution of the Bayesian-based Standard Ellipse Areas (SEAB) with 50%, 75%, and 95% credibility intervals for the three fishes (*Carassius auratus*, *Parabramis pekinensis*, and *Toxabramis swinhonis*) across different water periods. Crosses in red represent the area of the standard ellipse (SEAc). High. *P. pekinensis*: SEAB of *P. pekinensis* during high-water periods, high. *C. auratus*: SEAB of *C. auratus* during high-water periods, high. *T. swinhonis*: SEAB of *T. swinhonis* during high-water periods, low. *P. pekinensis*: SEAB of *P. pekinensis* during low-water periods, low. *C. auratus*: SEAB of *C. auratus* during low-water periods, low. *T. swinhonis*: SEAB of *T. swinhonis* during low-water periods, mid. *P. pekinensis*: SEAB of *P. pekinensis* during mid-water periods, mid. *C. auratus*: SEAB of *C. auratus* during mid-water periods, mid. *T. swinhonis*: SEAB of *T. swinhonis* during mid-water periods. Red x: the estimated SEAc, black dots: SEAB mode.

Despite the overall similarity in isotopic niches, with mean values of −26.4928‰ in δ^13^C and 15.9277‰ in δ^15^N, SIBER outputs revealed general segregation based on SEAc across different water periods (Figure 9 and Figure 10). Notably, *P. pekinensis* and *T. swinhonis* had their largest SEAc values during high-water periods (SEAc = 5.2108‰^2^ and 2.0002‰^2^ for *P. pekinensis* and *T. swinhonis*), whereas *C. auratus* demonstrated a narrow SEAc (1.2957‰^2^). Conversely, in low-water periods, *C. auratus* displayed its highest SEAc (2.4011‰^2^), while *P. pekinensis* and *T. swinhonis* narrowed its SEAc (3.1516‰^2^ and 1.1604‰^2^). Niche overlap analysis revealed that all species exhibited some degree of isotopic niche overlap across water periods, with lowest overlap during high-water periods and highest during low-water periods, except for the highest overlap between *C. auratus* and *T. swinhonis* during mid-water periods.

A Bayesian mixing model was employed to assess the contributions of primary carbon sources to the diets of the three fish species under study (Table 3). The results revealed that POM was the predominant dietary component for *P. pekinensis*, *C. auratus* and *T. swinhonis* in Hongze lake, contributing up to 76.52%, 80.25%, and 81.31%, with 95% credibility intervals of 76.42–76.62%, 80.15–80.34%, and 81.21–81.41%, respectively. SOM contributed 16.87% (16.73–17.00%) to *P. pekinensis*, 13.88% (13.76–14.01%) to *C. auratus*, and 13.22% (13.10–13.34%) to *T. swinhonis*, while macrophyte contributed 6.61% (6.52–6.71%) to *P. pekinensis*, 5.87% (5.78–5.96%) to *C. auratus* and 5.47% (5.39–5.55%) to *T. swinhonis*. During mid-water periods and low-water periods, POM contributed more than 75% to the diets for all three species, while the contributions of SOM and macrophyte were relatively low, ranging from 2.01% to 14.61% and 4.10% to 21.17%, respectively. During high-water periods, however, the contribution of POM to the diets decreased to 55.79–65.02%, with a corresponding significant increase in the contribution of SOM (20.93–26.82%) and a steadily rise in the contribution of macrophyte (14.05–17.39%).

## 4. Discussion

The study presented an isotopic analysis to assess TPs, niche widths and overlaps, and the contributions of primary carbon sources to the diets of three fish species, aiming to elucidate interspecific trophic interactions and coexistence mechanisms under off-seasonal water level regulation in Hongze Lake. The findings indicated that all three fish species generally occupied similar mid-level TPs across different water periods, although *P. pekinensis* exhibited a slightly lower TP compared to *T. swinhonis* and *C. auratus*. Notably, *P. pekinensis* and *T. swinhonis* exhibited broader niche width (SEAc) during high-water periods, whereas *C. auratus* reached its maximum SEAc during low-water period, suggesting trophic partitioning among these species. The high trophic overlap among *C. auratus* and *T. swinhonis* indicates a shared resource base and potential competition. All species exhibited some degree of isotopic niche overlap across water periods, with lowest overlap during high-water periods and highest during low-water periods, except for the highest overlap between *C. auratus* and *T. swinhonis* during mid-water periods. Finally, a Bayesian mixing model identified POM as the predominant dietary component for all three fish species. However, from mid-water periods to high-water periods, the contribution of POM decreased from 80.6–84.65% to 55.79–64.24%, with a corresponding significant increase in the contribution of SOM, and subsequently, macrophytes. This shift illustrates a differential utilization of primary carbon sources across water periods.

### 4.1. δ^13^C and δ^15^N Characteristics of Primary Carbon Sources and Three Fish Species in Hongze Lake

The present study identified notable variations in the δ^13^C values among primary carbon sources, while δ^15^N values showed no significant differences. In particular, the δ^13^C of POM (−26.29 ± 3.37‰) was significantly lower compared to that of SOM (−20.82 ± 4.59‰) and macrophyte (−19.70 ± 3.36‰). The observed range in δ^13^C values reflected the diversity of food sources within the food web, with a broader range indicating a greater variety of initial food sources and a more complex the food web structure in Hongze lake [47]. Although similar δ^13^C ranges of carbon resources between water periods, the significant differences in primary carbon source δ^13^C values suggest that water-level fluctuations due to water diversion may impact the lake’s trophic structure. Studies on lakes in the Yangtze basin, such as Poyang, Dongting, and Tian-e-Zhou, have demonstrated similar findings, with food webs influenced by changes in basal food sources due to hydrological alterations [48,49,50]. This study further revealed that contributions of these carbon sources to fish diets varied across water periods, alongside δ^15^N enrichment, enhancing the observed isotopic differentiation among species.

The present study revealed that the isotopic signatures of *P. pekinensis* were significantly different from those of *C. auratus* and *T. swinhonis*, while no significant difference was detected between *C. auratus* and *T. swinhonis*. This is likely linked to their respective diets. *C. auratus* is a typical omnivore [31], which fed on zooplankton, plant debris, phytoplankton, benthos in surrounding environment [51]. *T. swinhonis*, generally classified as a zooplanktivore [31], has a similar dietary profile (Hu et al., 2019), which may explain their isotopic similarity. In contrast, *P. pekinensis* primarily an herbivore and primary consumer, favors aquatic plants [52] but also consumes phytoplankton in winter and artificial feed in ponds [53], demonstrating dietary flexibility. With the decline in submerged macrophytes in Hongze Lake since the 1980s [54], the present study found a lower contribution of macrophytes (6.61%) to the diet of *P. pekinensis*, indicating a greater reliance on phytoplankton. Nonetheless, *P. pekinensis* retained the highest macrophytes contribution among the three species, resulting in elevated δ^13^C values relative to *C. auratus* and *T. swinhonis*. Additionally, *P. pekinensis*, as a primary consumer, showed weaker δ^15^N enrichment, yielding lower δ^15^N values relative to the other two species. Interestingly, δ^15^N values for all three species were higher during mid-water periods than low-water periods and high-water periods, likely due to seasonal shifts in plant food availability. Previous studies have reported that artificial water level fluctuations adversely impact macrophytes [55], with their biomass and abundance typically lower in winter than in other seasons [35]. Concurrently, phytoplankton biomass declines during winter due to water diversion-induced dilution effects [56]. Such resource shifts likely drive omnivorous fish to increase animal food consumption in winter, leading to relatively higher δ^15^N values. This inference is further supported by observed variations in macrophytes contributions to fish diets across water periods in the present study.

### 4.2. Trophic Position Variations of the Three Fish Species Across Different Water Periods

TP variations are influenced by a range of abiotic and biotic factors, including prey availability, diet composition, and hydrologic stability [57]. The three fish species examined—*P. pekinensis*, *C. auratus*, and *T. swinhonis*—are widely distributed and abundant in Hongze Lake [28]. Our results revealed that the three fish species exhibited similar mid-level TPs, although *P. pekinensis* exhibited a slightly lower TP compared to *C. auratus* and *T. swinhonis*. Similar findings align with previous research on these species (2.29–3.63 TPs) [32,48,50]. According to Woodland, et al. [58], intermediate consumers, often omnivorous, tend to dominate overall biomass in aquatic food webs, a hypothesis that holds for the relevance of TP and abundance of the three fish species observed here.

With omnivorous feeding habits, mid-level TP fishes also display flexible feeding strategies that allows species to exploit available food resources, especially in environment with fluctuating resource availability [59]. TP fluctuations are often tied to nutrient availability shifts. Surprisingly, there were no statistically significant differences in TP of three fish species across water periods in the present study (*p* > 0.05), despite a general decline in the TPs from mid-water periods to high-water periods. This outcome contrasts with findings reporting significant seasonal TP shifts in tropical floodplain omnivores [60,61]. Omnivores consuming more invertebrates during wet seasons would exhibit elevated TPs [60], while certain species, such as *Brycon* spp., may switch to fruits and seeds during wet periods, thereby lowering their TPs [8]. However, TP stability across seasons is also common among omnivorous fish species in dynamic ecosystems [48,62]. For instance, in Dongting Lake, 4 of 15 fish species (including *C. auratus*) showed no significant TP variation across seasons [50], while 25 of 36 species in Poyang Lake displayed similar stability [49]. Compared to herbivores, insectivores, and planktivores, omnivores in Poyang Lake exhibited milder TP variations across water periods [49]. Maintaining a consistent trophic position year-round is possible if fish consume similar prey types or a diverse, yet stable, array of prey [8,60]. Feeding on different but trophically similar prey as resources fluctuate spatially and temporally also contributes to stable TP [62]. Due to their broad dietary flexibility [63], omnivores also suffer less from water level fluctuation, resulting in only slight differences in their trophic positions between the dry and wet seasons [50]. Additionally, TP changes are minimal across similar body sizes [62], which was also reflected in our findings where body sizes remained consistent across water periods, likely contributing to the absence of significant TP variations.

### 4.3. Feeding Strategies of Three Fish Species Under Off-Seasonal Water Level Regulations

Niche theory is frequently employed to elucidate the mechanisms of coexistence and competition among species, with niche width and niche overlap playing crucial roles in understanding the status, role and interspecific relationships of species within a community [22]. Understanding how niches are partitioned and how interspecific competition affects niche width is critical, as these factors determine species’ ability to cope with water level fluctuations. In the present study, observed differences in niche width, as represented by SEAc, among the three fish species indicate a degree of niche partitioning. This aligns with the Optimal Foraging Theory [17], where subordinate foragers like *P. pekinensis* tend to diversify their diets under competition with dominant species, such as *C. auratus* and *T. swinhonis* during high-water periods. Similar patterns have been observed in other ecosystems, such as in sympatric sharks, where the subordinate species *Carcharhinus melanopterus* (Quoy and Gaimard, 1824) exhibits broader habitat use and feeding niches than competitively dominant *Negaprion acutidens* (Rüppell, 1837) [64]. In the current study, during high-water periods, the SEAc of *P. pekinensis* and *T. swinhonis* were higher than the other water periods, whereas the SEAc of *C. auratus* exhibited the opposite trend. During high-water periods, fishes have access to more diverse habitats and novel food sources after accessing inundated areas and including terrestrial food sources in their diets, resulting in the larger niche width [65,66]. For instance, terrestrial resources significantly contribute to the diet of *Moenkhausia forestii* during the high-water periods [24]. In contrast, low-water periods typically reduce the available habitats and concentrate food resources, leading to more specialized feeding strategy [67,68]. This is consistent with our observation in the SEAc of *P. pekinensis* and *T. swinhonis*. A similar trend has been reported for piranha species in Amazonian floodplain lakes [69]. In Hongze Lake, diet biomass for *P. pekinensis* and *T. swinhonis*, including macrophytes, phytoplankton, and zooplankton, is often scarce during high-water periods (winter and early spring) due to unnatural high water level and low water temperature, but becomes abundant during low-water periods (summer) because of limited space and high productivity [33,34,35]. A generally larger SEAc for *P. pekinensis* and *T. swinhonis* during high-water periods compared to low-water periods indicates that these species expanded their resource utilization by consuming a broader range of prey items once they could access fluctuating water zones [66]. The narrow niche widths of *P. pekinensis* and *T. swinhonis* during low-water periods suggest that they may target most readily available or profitable prey types [30,70], leading to reduced SEAc of them. These results are in accordance with the Optimal Foraging Theory and consistent with previous findings on *P. pekinensis* along the Three Gorges Dam [71], *Moenkhausia forestii* in a floodplain lake of the Upper Paraná River [24], *Channa micropeltes*, *Hemibagrus spilopterus*, *Notopterus notopterus*, and *Pangasius larnaudii* [66], suggesting that both *P. pekinensis* and *T. swinhonis* are optimal forgers.

In contrast, *C. auratus*, characterized by broad dietary flexibility [51], exhibits a different pattern in SEAc. During low-water periods, due to reduced area, water volume, depth and connectivity between aquatic environments, habitats and resources are more concentrated. In such conditions, sympatric competitors such as *T. swinhonis* may target most readily available or profitable prey of *C. auratus* [67,68], which push *C. auratus* to include alternative and less profitable food resources in their diets. This dietary flexibility could result in a wider niche (larger SEAc) of *C. auratus* during low-water periods, as the species takes advantage of a broader array of food resources in response to limited availability of its preferred prey [31]. Conversely, during high-water periods, *C. auratus* may narrow its dietary and focus on the most optimal prey due to decreased competition arising from the expanded habitats and diverse food resources. This specialization during high-water periods could lead to a narrower niche width (smaller SEAc). The responses of *C. auratus* to off-seasonal water level regulation are consistent with predictions of classical competition theory and aligns with *C. auratus* in Poyang Lake [20] and along the Three Gorges Dam [71].

### 4.4. Coexistence Mechanism of Three Fish Species Under Off-Seasonal Water Level Regulations and Its Implications

The coexistence pattern of the three fish species in Hongze Lake is primarily driven by their adaptive responses to off-seasonal water level regulations, as revealed by a shifting reliance on carbon sources. Specifically, the transition from POM to SOM and macrophytes underscores the flexibility of these species in adapting to fluctuating environments. In fact, adaptive reactions to the manifestations of factors have been previously known in various vertebrates [72,73]. This once again confirms their presence in such vertebrates as fish in lakes. As niche width changed with water level fluctuations, niche overlaps among the three fish species also vary across water periods. Our results reveal that the trophic overlaps between *P. pekinensis* and the other two species are lowest during high-water periods but generally highest during low-water periods. During high-water periods, increased habitat availability and diversified yet reduced food resources result in generally low interspecific niche overlap between *P. pekinensis* and the others. The competitive exclusion principle suggests that species competing for the same resources can coexist only through niche differentiation [74,75], typically achieved by partitioning food and space [76,77]. The decreased overlap during high-water periods indicates that the coexistence of *P. pekinensis* with *C. auratus* and *T. swinhonis*. is primarily achieved through trophic niche segregation. Similar phenomena have been widely found in Amazonian floodplain lakes, including piranha species [69] and *Moenkhausia* fishes [78]. Conversely, during low-water periods, the receding water concentrates productive food resources, providing *P. pekinensis* with opportunities to consume preferred resources, such as macrophytes and phytoplankton. However, this period also intensifies competition with *C. auratus* and *T. swinhonis*, resulting in a narrower niche and increased overlap. While the coexistence of *P. pekinensis* with *C. auratus* and *T. swinhonis* is likely supported by sharing abundant trophic resources, as seen in sympatric snapper species [79], the high niche overlap during low-water periods might cause intense competition and potentially degradation of *P. pekinensis* populations. Thus, off-seasonal water level regulation in Hongze Lake has profound influences on the resource utilization of *P. pekinensis.*

Comparatively, the trophic overlaps between *C. auratus* and *T. swinhonis* during three water periods were higher than the others and peaked during mid-water periods, indicating a shared resource base and intense competition under off-seasonal water level regulation. As a dominant species with optimal forging behavior, *T. swinhonis* would consume more preferred resources when food resources are abundant and compete aggressively for preferred resources when food resources are scarce. Meanwhile, *C. auratus*, as a generalist feeder, would broaden its diet or consume alternative food resources to cope with competition from *T. swinhonis* and other species. It is widely accepted that omnivory plays a crucial role in minimizing competition for trophic space, as the ability to consume a wider range of food sources enhances resilience to resource scarcity and environmental changes [65,71,77,80,81]. This adaptability may make omnivores the most successful under future environmental changes, and enhance their ability to coexist in a fluctuating environment [59,60,62]. In spite of this flexibility facilitates the coexistence of *C. auratus* with *T. swinhonis*, continuous or increased competitive pressure exerted by *T. swinhonis* may affect the resource utilization of *C. auratus*. Consequently, the competition interactions between *T. swinhonis* and *C. auratus* cannot be not ignored, and trophic interactions and population dynamics of these fish species under water diversion should be monitored continually.

## 5. Conclusions

This study highlights the significant role of off-seasonal water level regulations in shaping the trophic dynamics and coexistence strategies of fish species in Hongze Lake. Despite occupying similar mid-level trophic positions, *P. pekinensis*, *C. auratus*, and *T. swinhonis* exhibit distinct niche adaptations across different water periods. During high-water periods, *P. pekinensis* and *T. swinhonis* exploit broader niches due to expanded habitat and diverse food resources, while *C. auratus* relies on a narrower diet. In contrast, during low-water periods, *C. auratus* adopts a generalist feeding strategy, expanding its niche, while *P. pekinensis* and *T. swinhonis*, as optimal forgers, reduce their isotopic niche widths. These findings demonstrate that dynamic resource partitioning during high-water periods and resource sharing during low-water periods promote the coexistence of these species. Given that off-seasonal water level regulation intensify interspecific competition, continuous monitoring of trophic interactions and population dynamics among these fish species is crucial. These insights provide valuable guidance for refining water management policies and developing sustainable fishery management strategies for Hongze Lake and other water-level-regulated systems.

## Figures and Tables

**Table 1:** Physico-chemical characteristics (mean value ± sd) of water in Hongze Lake during three water periods.

Water Periods	Regions	Temperature °C	Water Depth (m)	Transparency (cm)	Total Phosphorus (mg/L)	Total Nitrogen (mg/L)	Chlorophyll a (μg/L)	pH	Dissolved Oxygen (mg/L)	Conductivity μS/cm
mid-water	Eastern	9.60 ± 0.66	3.04 ± 0.60	27.80 ± 7.92	0.046 ± 0.019	2.089 ± 0.383	18.549 ± 6.560	8.59 ± 0.37	12.620 ± 0.525	446.62 ± 16.73
	Northern	10.98 ± 0.22	2.85 ± 0.13	26.75 ± 5.19	0.050 ± 0.013	1.183 ± 0.166	30.340 ± 19.374	8.85 ± 0.24	12.713 ± 1.248	512.25 ± 36.56
	Southern	10.33 ± 0.45	2.63 ± 0.67	56.67 ± 11.93	0.034 ± 0.004	1.928 ± 0.615	12.169 ± 2.054	8.89 ± 0.03	13.017 ± 0.116	427.33 ± 33.41
	Western	9.27 ± 0.56	2.50 ± 0.10	22.80 ± 11.28	0.070 ± 0.027	1.384 ± 0.300	22.693 ± 11.163	7.79 ± 0.76	13.323 ± 0.171	511.75 ± 58.84
low-water	Eastern	30.64 ± 1.17	2.10 ± 0.27	13.25 ± 4.65	0.315 ± 0.492	2.379 ± 0.576	35.323 ± 9.081	9.39 ± 0.27	8.215 ± 1.344	706.13 ± 116.00
	Northern	30.40 ± 1.47	2.02 ± 0.27	16.15 ± 2.61	0.112 ± 0.033	2.113 ± 1.243	74.740 ± 22.375	8.32 ± 0.48	9.861 ± 1.601	742.85 ± 33.17
	Southern	32.30 ± 0.52	2.23 ± 0.78	20.00 ± 4.58	0.123 ± 0.036	2.396 ± 0.767	33.876 ± 22.507	9.07 ± 0.17	8.540 ± 2.691	583.00 ± 45.43
	Western	30.06 ± 2.22	1.91 ± 0.21	19.50 ± 5.67	0.142 ± 0.047	1.503 ± 0.501	59.595 ± 33.098	7.89 ± 1.38	9.466 ± 2.319	943.64 ± 132.57
high-water	Eastern	11.40 ± 1.36	3.20 ± 0.08	22.25 ± 12.69	0.166 ± 0.064	2.647 ± 1.148	6.907 ± 1.846	10.90 ± 1.10	12.758 ± 1.159	342.40 ± 83.25
	Northern	10.40 ± 0.21	2.82 ± 0.96	17.10 ± 4.91	0.089 ± 0.028	1.769 ± 0.632	7.624 ± 3.392	9.40 ± 1.43	13.349 ± 0.653	494.29 ± 85.34
	Southern	12.00 ± 0.71	3.35 ± 1.06	31.00 ± 4.24	0.105 ± 0.006	2.716 ± 0.270	2.631 ± 0.570	9.32 ± 0.70	12.555 ± 0.205	390.65 ± 34.30
	Western	10.91 ± 0.91	2.87 ± 0.38	17.11 ± 4.96	0.142 ± 0.071	2.168 ± 0.613	5.673 ± 3.316	10.33 ± 1.19	13.030 ± 0.780	438.70 ± 128.46

**Table 2:** Isotopic niche overlap among three fishes in Hongze Lake and across different water periods, based on the maximum likelihood fitted ellipses with 95% credibility intervals derived from Bayesian posterior distributions.

Predator 1—Predator 2	Pred. 1 (%)	95% CI	Pred. 2 (%)	95% CI	Overlap Between Pred. 1 and Pred. 2 (%)
In the whole lake					
*P. pekinensis*—*C. auratus*	23.3057	19.8693–25.6070	9.7826	7.4732–11.6734	28.79
*P. pekinensis*—*T. swinhonis*	23.3057	19.8693–25.6070	14.8831	11.7498–15.9053	33.79
*C. auratus*—*T. swinhonis*	9.7826	7.4732–11.6734	14.8831	11.7498–15.9053	39.52
Across different water periods					
*P. pekinensis*—*C. auratus* (mid-water periods)	20.4791	12.0613–24.6589	7.8633	16.3888–23.8486	23.63
*P. pekinensis*—*T. swinhonis* (mid-water periods)	20.4791	12.0613–24.6589	10.5111	21.8892–46.3491	26.28
*C. auratus—T. swinhonis* (mid-water periods)	7.8633	16.3888–23.8486	10.5111	21.8892–46.3491	34.75
*P. pekinensis*—*C. auratus* (low-water periods)	18.8702	6.2007–23.7846	14.3762	4.7764–10.2699	31.48
*P. pekinensis*—*T. swinhonis* (low-water periods)	18.8702	6.2007–23.7846	6.9476	6.2286–11.0645	26.91
*C. auratus*—*T. swinhonis* (low-water periods)	14.3762	4.7764–10.2699	6.9476	6.2286–11.0645	29.23
*P. pekinensis*—*C. auratus* (high-water periods)	31.1993	4.1066–8.3071	7.7581	5.8393–15.1646	19.91
*P. pekinensis*—*T. swinhonis* (high-water periods)	31.1993	4.1066–8.3071	11.9764	7.5546–17.3958	22.85
*C. auratus*—*T. swinhonis* (high-water periods)	7.7581	5.8393–15.1646	11.9764	7.5546–17.3958	27.23

Note: Pred. 1 indicates the percentage of the isotopic niche of “predator 1” that is overlapped by “predator 2”. Pred. 2 indicates the percentage of the isotopic niche of “predator 2” that is overlapped by the “predator 1”.

**Table 3:** Mean percentage contributions (95% credibility interval) of the primary carbon sources to the diets of three fishes in Hongze Lake and across different water periods, as analyzed using Bayesian stable isotope mixing model.

Food Resources	Contribution to Fish Species (%)		
	*Parabramis pekinensis*	*Carassius auratus*	*Toxabramis swinhonis*
In the whole lake			
SOM	16.87 (16.73–17.00)	13.88 (13.76–14.01)	13.22 (13.10–13.34)
POM	76.52 (76.42–76.62)	80.25 (80.15–80.34)	81.31 (81.21–81.41)
Macrophyte	6.61 (6.52–6.71)	5.87 (5.78–5.96)	5.47 (5.39–5.55)
Across different water periods			
SOM (mid-water)	14.61 (14.46–14.76)	11.25 (11.13–11.37)	12.94 (12.81–13.07)
POM (mid-water)	80.60 (80.47–80.72)	84.65 (84.55–84.75)	81.14 (81.04–81.24)
Macrophyte (mid-water)	4.79 (4.73–4.85)	4.10 (4.05–4.15)	5.92 (5.86–5.99)
SOM (low-water)	2.21 (2.18–2.25)	2.03 (01.99–02.07)	2.01 (1.98–2.05)
POM (low-water)	76.62 (76.39–76.84)	80.91 (80.71–81.11)	80.61 (80.41–80.81)
Macrophyte (low-water)	21.17 (20.93–21.41)	17.06 (16.85–17.27)	17.38 (17.17–17.59)
SOM (high-water)	26.82 (26.52–27.13)	20.93 (20.63–21.23)	21.20 (20.88–21.51)
POM (high-water)	55.79 (55.49–56.08)	65.02 (64.66–65.38)	64.24 (63.85–64.64)
Macrophyte (high-water)	17.39 (17.13–17.65)	14.05 (13.81–14.28)	14.56 (14.31–14.81)

## Data Availability

Data are available from the authors upon reasonable request.
